# Automated Biochemical, Morphological, and Organizational Assessment of Precancerous Changes from Endogenous Two-Photon Fluorescence Images

**DOI:** 10.1371/journal.pone.0024765

**Published:** 2011-09-09

**Authors:** Jonathan M. Levitt, Margaret E. McLaughlin-Drubin, Karl Münger, Irene Georgakoudi

**Affiliations:** 1 Department of Biomedical Engineering, Tufts University, Medford, Massachusetts, United States of America; 2 Department of Medicine, Brigham and Women's Hospital, Harvard Medical School, Boston, Massachusetts, United States of America; Université de Technologie de Compiègne, France

## Abstract

**Background:**

Multi-photon fluorescence microscopy techniques allow for non-invasive interrogation of live samples in their native environment. These methods are particularly appealing for identifying pre-cancers because they are sensitive to the early changes that occur on the microscopic scale and can provide additional information not available using conventional screening techniques.

**Methodology/Principal Findings:**

In this study, we developed novel automated approaches, which can be employed for the real-time analysis of two-photon fluorescence images, to non-invasively discriminate between normal and pre-cancerous/HPV-immortalized engineered tissues by concurrently assessing metabolic activity, morphology, organization, and keratin localization. Specifically, we found that the metabolic activity was significantly enhanced and more uniform throughout the depths of the HPV-immortalized epithelia, based on our extraction of the NADH and FAD fluorescence contributions. Furthermore, we were able to separate the keratin contribution from metabolic enzymes to improve the redox estimates and to use the keratin localization as a means to discriminate between tissue types. To assess morphology and organization, Fourier-based, power spectral density (PSD) approaches were employed. The nuclear size distribution throughout the epithelial depths was quantified by evaluating the variance of the corresponding spatial frequencies, which was found to be greater in the normal tissue compared to the HPV-immortalized tissues. The PSD was also used to calculate the Hurst parameter to identify the level of organization in the tissues, assuming a fractal model for the fluorescence intensity fluctuations within a field. We found the range of organization was greater in the normal tissue and closely related to the level of differentiation.

**Conclusions/Significance:**

A wealth of complementary morphological, biochemical and organizational tissue parameters can be extracted from high resolution images that are acquired based entirely on endogenous sources of contrast. They are promising diagnostic parameters for the non-invasive identification of early cancerous changes and could improve significantly diagnosis and treatment for numerous patients.

## Introduction

Cervical cancer is the second most common cancer in women, leading to over 309,000 deaths worldwide annually [1]. Accurate and early screening is essential, since when detected early, the survival rate of cervical cancer patients is almost 100% [2]. Typically, cervical cancer screening is performed using the Papanicolaou (Pap) test, during which cells are scraped from the cervix, fixed, stained and observed under magnification. If morphological abnormalities are detected, the cervix is further investigated using colposcopy, and biopsies are extracted from suspicious regions. These methods are time consuming, expensive, and have variable performance depending on the screener's level of expertise, which disproportionally affects developing nations. The microscopic scale at which these early changes occur point to the need for a higher resolution approach than standard colposcopy, such as optical, depth-resolved, high-resolution microscopy.

Specifically, two-photon excited fluorescence (TPEF) microscopy can be used to assess at least some of the standard histological hallmarks of pre-cancers, while observing cells in their native environment, where additional diagnostically useful parameters can be measured. For example, since cellular autofluorescence is predominantly confined to the cytoplasm, the nuclei are well defined by the absence of fluorescence and features such as nuclear size can be assessed to discriminate between normal and pre-cancerous tissue [3]. While researchers have used spatial domain segmentation approaches to quantify morphology, we have developed methods based on Fourier-domain analysis that can be implemented automatically to characterize *all* cells imaged within a field. Using these techniques we are also able to assess the sub-cellular organization [4], [5], which is largely overlooked during standard histopathological evaluation, but could provide highly sensitive indicators of early cancer [6]. Since the endogenous TPEF signals we examine predominantly emanate from NADH and FAD, our method is specifically sensitive to the level of mitochondrial organization. Endogenous fluorescence from these two key oxidative phosphorylation enzymes has been used as a sensitive marker of oxygen consumption and metabolism [7]. Studies have reported an increase in metabolic activity associated with cancer progression by assessment of ATP via enzymatic analysis [8] and of the redox ratio (the ratio of FAD to NADH) via optical techniques [9], [10]. It is worth noting that NADPH, a molecule not involved in energy production, has identical excitation and emission spectra to NADH. However, the fluorescence quantum yield of NADPH is substantially lower than that of NADH, and hence it has little effect on quantitative calculations [11], [12]. The spectrally inseparable signals of NADH and NADPH will be referred to as NAD(P)H from here on.

The goal of this study was to develop a quantitative method to discriminate between normal and pre-cancerous tissue based on the combination of biochemical and morphological parameters obtained through non-invasive, optical, endogenous TPEF measurements on model tissues. Specifically, we show how metabolism, morphology, and sub-cellular organization can be assessed to identify changes associated with human papillomavirus (HPV) oncogene expression. HPV is the cause of over 90% of cervical cancers and of a significant percentage of cancers in the oral cavity and anogenital tract [1], [13]. However, we anticipate that the optical biomarkers we discovered and describe here are more broadly applicable to early cancer detection, as the molecular pathways dysregulated by HPV oncogene expression, i.e. the p53 and retinoblastoma (pRB) tumor suppressor pathways, are commonly dysfunctional in a broad range of epithelial cancers. Furthermore, the methods we present are automated and based on non-computationally-intensive calculations, which can be implemented in real time and are suitable for ultimate implementation in a clinical setting.

## Materials and Methods

### Engineered Tissues

As a model for high-grade HPV-associated precancerous lesions, we used the HKc/DR cell line. This clonal cell line was established by immortalization of human foreskin keratinocytes derived from a single donor by expression of a head-to-tail dimer of the full-length HPV16 genome followed by selection for TGF-β and differentiation resistance[14], [15]. These cells are a model for high-grade premalignant lesions in that they are non-tumorigenic and mRNA expression analyses have shown that they are similar to cervical carcinoma cells [16]. As a control, we used primary human foreskin keratinocytes isolated from neonates and grown in serum free medium as described elsewhere [17] Isolation of human cells was approved by the Partners Human Research committee at the Brigham and Woman's Hospital, IRB # 2006-P-000466/1. The IRB waived the need for consent as this procedure did not meet the definition of human subject's research, and, therefore, didn't require informed consent because we used unidentified foreskins that would be otherwise discarded and we didn't intervene or interact with a living person in order to acquire them. Organotypic tissues were constructed from the two cell types using established protocols [18]. In brief, a dermal equivalent of J2 3T3 fibroblasts and rat tail collagen (4 mg/ml) was allowed to gel for 12 hours before normal or HPV-immortalized cells were seeded on top. Once the cells reached confluence the tissue structures were raised to the air-liquid interface and the cells were allowed to differentiate over 10 days before harvesting.

### Two-Photon Imaging

We acquired TPEF and second harmonic generation (SHG) images using a Leica TCS-SP2 microscope equipped with a Mai Tai, Ti:Saphire laser (Spectra Physics). Samples were excited through a 63×, 1.2NA water immersion objective at 755, 800, and 860 nm with average powers of 15–20 mW. For spectral acquisition, we acquired 30 images corresponding to 20 nm bandwidths over the range of 400–700 nm. Spectra were interpolated to 1 nm resolution and smoothed using a 30 point moving average window. Depth stacks were acquired at 1 µm depth increments and light was collected using two non-descanned PMTs through a 700 nm short pass filter and either a 455±35 nm (HQ455 72m2p, Chroma) or a 525±25 nm bandpass filter (HQ525 50m, Chroma). Six frames were averaged at each depth to reduce noise. SHG from collagen was excited using 800 nm light and detected in the backwards direction through a 400±10 nm filter. To compare images taken using different PMT settings and excitation powers, we measured a range of fluorescein concentrations at each setting to relate the pixel intensity to a relative fluorophore concentration [19]. Images were processed using MATLAB (Mathworks) and Leica Confocal Software (Leica).

### Power Spectral Density Analysis

To characterize the spatial composition of the images, we calculated the power spectral density (PSD) as described previously [4]. We fit the PSD slopes over frequencies greater than 10 µm^−1^ to eliminate the artifacts that are due to the strong nuclear contribution at the lower frequencies. The range of organization in a given sample was calculated by subtracting the minimum from the maximum Hurst value throughout the epithelium. The variance of the PSD was calculated at each frequency across the transverse direction of the tissues to identify the relative contribution of specifically sized objects throughout the epithelial depth. To account for the decrease in power at greater depths, we report the variance parameter normalized to the variance at the highest frequency, which corresponds to the noise floor.

### Spectral Analysis

To assess the relative contributions from the different fluorophores in the tissues, we decomposed the emission spectra using an alternating least-squares (ALS) method with non-negativity constraints (PLS Toolbox for MATLAB, Eigenvector).

To analyze the TPEF emission spectra, we assumed that major contributions originated from NAD(P)H, FAD, and keratin at excitation wavelengths of 755, 800, and 860. The keratin emission components, obtained from spectral measurements of human skin extracted from the hand, foot, and arm, were found to be consistent with literature values and varied with excitation wavelength [20]. These excitation-dependent component spectra were used in the subsequent analysis of 81 emission spectra, obtained at various depths, from both tissue types to extract the emission profiles of two additional components needed to describe the composition of the spectra (unmodeled variance <0.10) using the ALS algorithm. The spectra of the two extracted components were consistent with NAD(P)H and FAD emission and independent excitation wavelength, consistent with our measurements of pure FAD and bound NADH prepared according to an established protocol [21]. Once we identified the spectral components of keratin, NAD(P)H, and FAD, we fixed them (Fig. S1) and used the PLS toolbox to find the relative contribution of the three fluorophores to a given emission spectrum.

### Image Calibration and Processing

The detected pixel intensities were converted to an equivalent fluorescein concentration to be able to compare images obtained at different acquisition settings. Then, a simple thresholding procedure was implemented to identify keratin(+) and keratin (−) regions.

Since the pixel intensity within an image is influenced by a number of systematic factors and not necessarily linearly related to fluence rate, we converted the intensity values to a corresponding fluorescein concentration. At each acquisition setting we measured a range of fluorescence concentrations to create a standard curve. The standard curve was used to convert the pixel intensities corresponding to NAD(P)H, FAD, and keratin fluorescence into a normalized concentration estimate so that we could accurately compare images that were obtained at different acquisition settings and better assess metabolic activity.

### Redox Calculation for Metabolic Assessment

The redox values were calculated by taking the ratio of the concentration estimate in the 525 nm channel at 860 nm (FAD) contribution to the concentration estimate in the 455 nm channel at 755 nm (NAD(P)H) contribution after removing effects from keratin via thresholding. The mean redox ratios were calculated over the entire depths of the tissues. To evaluate the consistency of the redox ratio throughout depths, we calculated the redox variability, which is the mean change in redox ratio per 1 µm depth increment throughout the tissues. We also assessed the keratin localization, which was defined as the percent of the epithelial tissue volume from the tissue surface where 90% of the total keratin resided, as computed from the thresholded images. Therefore, high levels of keratin localization correspond to lower percentage values.

### Keratin separation from NAD(P)H and FAD via thresholding

Keratin is not involved in metabolic processes and therefore we removed the contribution by use of binary masks, which were generated by applying a threshold to a matrix that represented the sum of the concentration estimates from the 455 nm and 525 nm channels at 755 and 860 nm excitation, respectively. Since the emission from keratin is much stronger than NAD(P)H and FAD, we found two distinct intensity populations within the sum matrix which allowed for a manual choice of a threshold value which remained fixed throughout this study.

### Statistical Analysis

All statistical analyses of single parameters were performed using a two-tailed, T-test. The diagnostic power of the combined parameters was assessed via multivariate linear regression with Statistical Analysis Software (SAS Institute). We evaluated the different combinations of parameters by assessing the R^2^ value, which corresponded to the ability of the variables to predict the tissue type. To determine which variables held the greatest diagnostic value we evaluated the type III error.

## Results

### Histological Validation of Optical Biopsy

**Figure 1:**
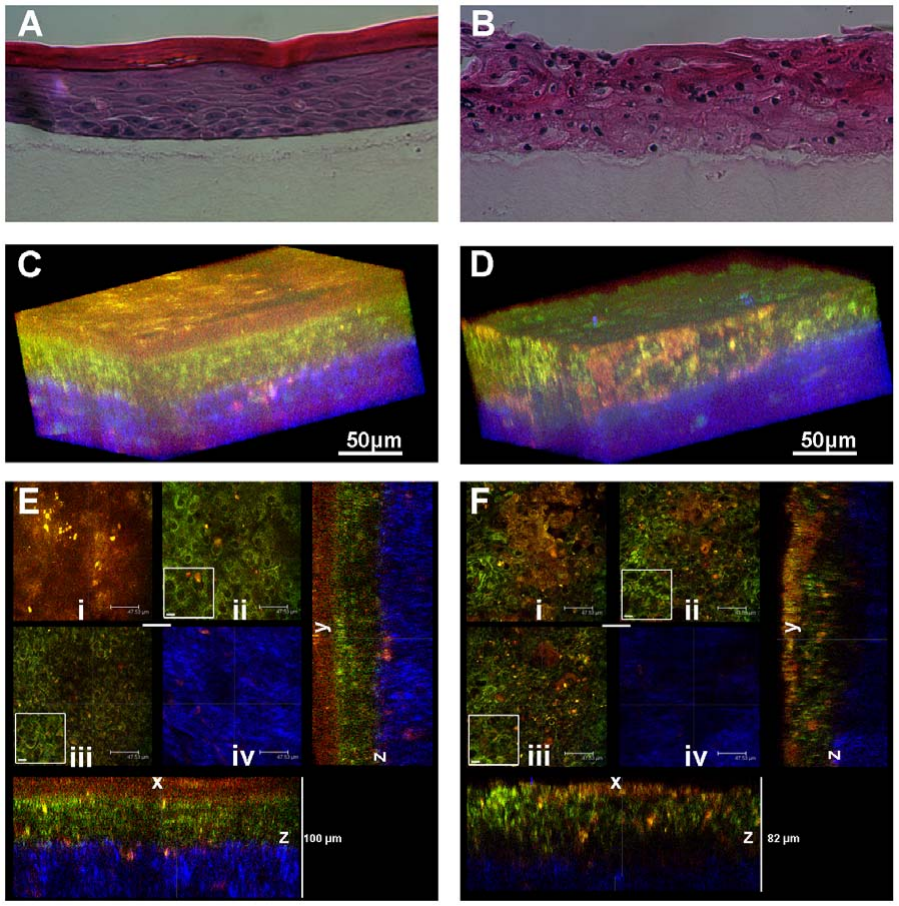
Histology and fluorescence based images of tissue. Normal (left column) and HPV- immortalized tissues (right column). Histological hematoxylin and eosin staining (*a–b*). False-color, depth-resolved TPEF and SHG images (*c–f*) from tissue excited at 755 nm and 800 nm. Green is fluorescence in the 455 nm channel (from NADH and keratin), while red is signal from the 525 nm channel (from FAD and keratin), acquired at 755 nm excitation. Blue represents 400 nm SHG detected at 800 nm excitation. Three-dimensional reconstructions were created from the fluorescence images (*c–d*). Corresponding depth images are shown in (*e–f*) with increasing depth from i to iv with zoomed insets to highlight the cell sizes. The scale bar represents 47.5 µm in the images and 10 µm in the zoomed insets. The transverse views of the normal and HPV-immortalized tissues (zx & zy) are shown with scale bars of 100 µm and 82 µm, respectively.

TPEF imaging of epithelial tissues, relying entirely on endogenous signals, yields images of high morphological detail, comparable to that of microscopic images acquired from excised tissues. Shown in Figure 1 are images acquired from engineered tissues containing primary human keratinocytes (left column) and HPV16-immortalized keratinocytes (right column) via histological staining (A and B) and optical depth-sectioning achieved with TPEF and second harmonic generation (SHG) imaging (C–F). Hematoxylin and eosin absorption provide optical contrast for the histology sections, while the source of contrast in the TPEF and SHG images (C–F) is fluorescence from NADH, FAD, and keratin (755 nm excitation), and scattering from collagen (800 nm excitation), respectively. In the normal tissue, we note a high level of differentiation, and variation in nuclear and cell size from the basal layer to the cornified layer, where a high level of keratin fluorescence is observed (Fig. 1A). In the tissue equivalents generated with HPV-immortalized cells, we detect a low level of cellular differentiation and consistent nuclear sizes throughout the various depths, as well as the absence of a keratinized uppermost layer (Fig. 1B).

The morphological trends observed in the histological sections are also evident in the 3-D reconstructions of the tissues (Fig. 1C–D), generated from high-resolution optical depth sections (Fig. 1E–F). From these reconstructions, the collagen border, epithelial thickness, and degree of stratification can be easily observed as in the histological images. Differences in keratin expression between the two tissue types are much more obvious in the intrinsic optical images than in the H&E stained sections, since keratin is highly autofluorescent.

### Morphological and Organizational Quantification

**Figure 2:**
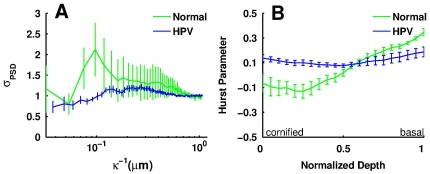
Morphological and organizational assessment. The (a) variation of the PSD as a function of frequency and the (b) Hurst parameter, as a function of normalized depth for the two tissue types, from the basal (value of 0) to the upper most tissue layer (value of 1).

To assess the morphology and organization of the depth-resolved fluorescence images, we rely on Fourier domain-based analysis. We specifically use the power spectral density (PSD), i.e. the squared amplitude of the two-dimensional Fourier transform of TPEF images dominated by mitochondrial NAD(P)H. This analysis was performed on five independent samples from each tissue type. To quantify the degree of spatial feature homogeneity in the samples, we calculate the variance of the PSD at each spatial frequency through the depth of the tissues (Fig. 2A). We observe that the PSD variance is most sensitive to changes in nuclear size as its highest value corresponds to spatial frequencies around 0.1 µm^−1^ (features on the order of 10 µm). Since there is a clear gradient of nuclear size in the normal tissue in contrast to the uniform distribution of nuclear sizes in the HPV tissues, we can use the spatial frequency variance at 0.1 µm^−1^ as a means to morphologically discriminate between tissue types. We find this parameter to differ (p<0.10) between the normal and HPV tissues with mean values of 2.02±0.828 and 0.931±0.0375, respectively.

To assess the level of mitochondrial organization in the tissues, we calculate from the PSDs the Hurst parameter as a function of epithelial tissue depth [4] (Fig. 2B). The Hurst parameter describes the fractional Brownian spatial features and consequently the level of self-correlation in an image [22]. As the NAD(P)H fluorescence we detect emanates primarily from mitochondria, the Hurst parameter is related to the manner in which mitochondria organize. Since individual mitochondria have characteristic sizes on the order of hundreds of nanometers, and are therefore smaller than the resolution of the images we acquire, the Hurst parameter is sensitive to mitochondrial networks and their degree of self-correlation (see Fig. 1 E&F insets). We find that the level of mitochondrial organization in the normal tissue decreases with the loss of cellular differentiation, since the Hurst parameter values for the basal/suprabasal layers approach 0.5, the value corresponding to Brownian behavior. In contrast to the normal tissue, the HPV immortalized tissues exhibit organization similar to that detected at the basal/suprabasal layers of the normal tissue with very little variation as a function of depth, thus reaffirming the correlation between differentiation status and mitochondrial organization [4]. The range of Hurst parameters encountered across the epithelial depth is one metric of this phenomenon, and, as expected, we find statistically different (p<0.05) Hurst ranges for the normal and HPV immortalized tissue groups with mean values of 0.543±0.183 and 0.190±0.0462, respectively.

### Spectral Analysis for Metabolic Assessment

**Figure 3:**
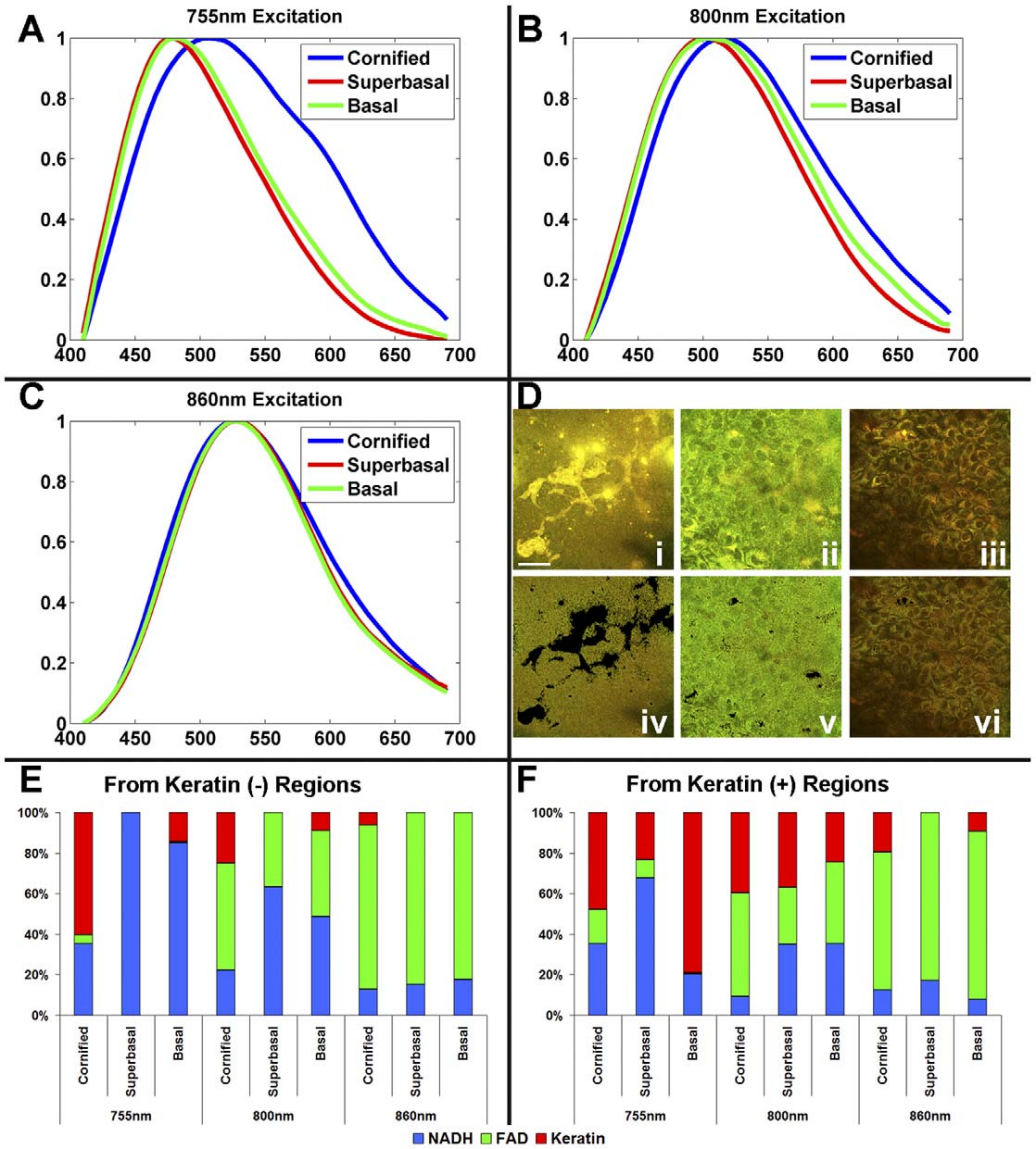
Spectra analysis from normal tissue. Fluorescence emission from 755 nm (A), 800 nm (B), and 860 nm (C) excitation at the cornified (blue), suprabasal (red), and basal (green) layers and the corresponding fields (D) before (i–iii) and after (iv–vi) thresholding to identify the keratin(+) regions (scale bar – 47.5 µm). Spectral decomposition was performed on the keratin(−) (E) and keratin(+) (F) areas to extract the relative contributions of NAD(P)H, FAD and keratin.

**Figure 4:**
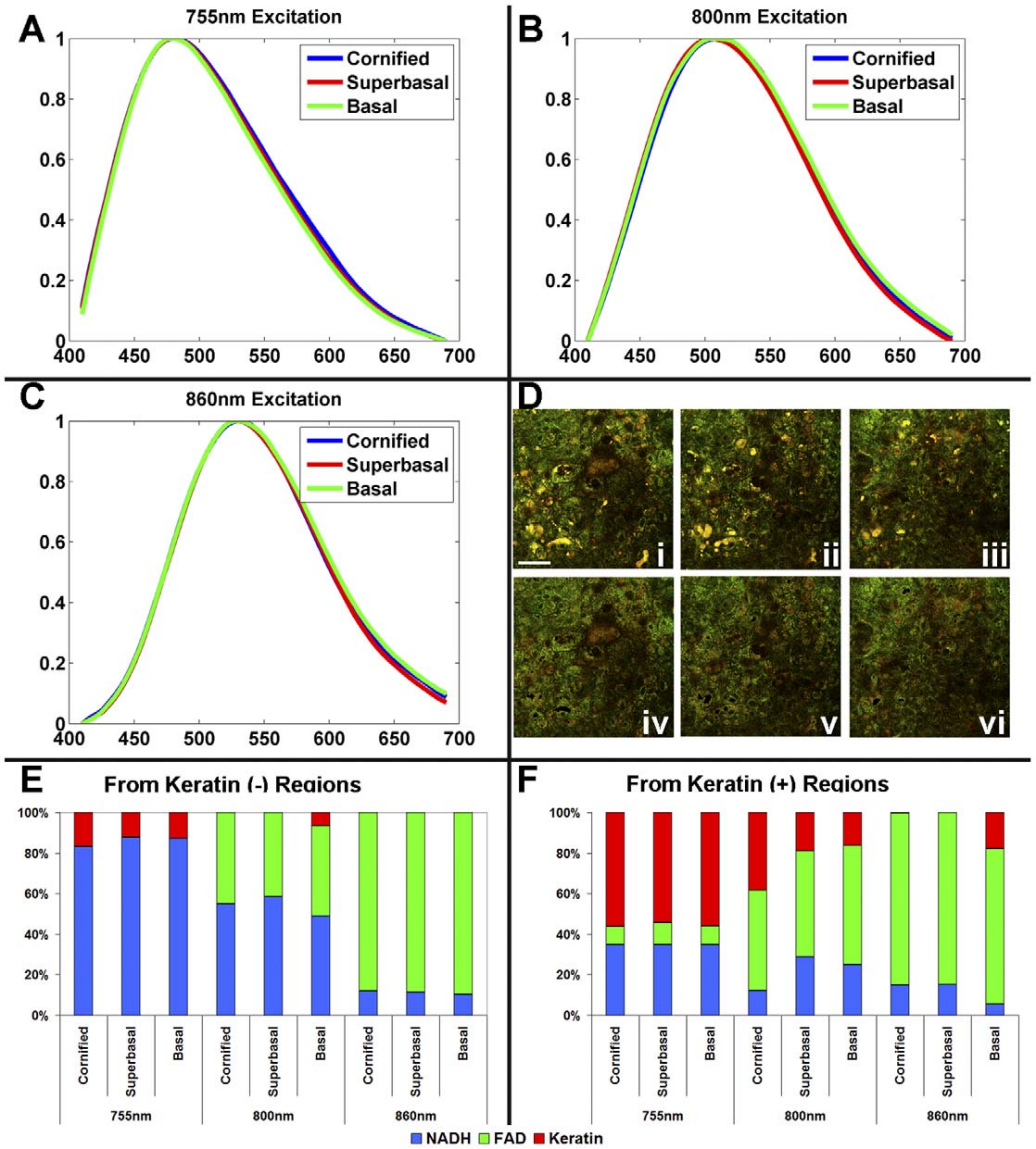
Spectral analysis from HPV immortalized tissues. Fluorescence emission from 755 nm (A), 800 nm (B), and 860 nm (C) excitation at the cornified (blue), suprabasal (red), and basal (green) layers and the corresponding fields (D) before (i–iii) and after (iv–vi) thresholding to identify the keratin(+) regions (scale bar – 47.5 µm). Spectral decomposition was performed on the keratin(−) (E) and keratin(+) (F) intensity areas to extract the relative contributions of NAD(P)H, FAD and keratin.

One of the main advantages of endogenous TPEF imaging is the capability to extract quantitative biochemical information about the samples under investigation. Spectral decomposition approaches offer a sensitive way of achieving this goal, especially in initial studies, when the number and spectral excitation/emission profiles of the relevant components may not be known. Peak-normalized fluorescence emission spectra from normal and HPV-immortalized tissues are shown in Figures 3 and 4, respectively, acquired at 755 nm (A), 800 nm (B) and 860 nm (C) excitation, corresponding to fields within the cornified D(i), suprabasal D(ii), and basal D(iii) layers. Spectral decomposition was achieved using an alternating least squares (ALS) minimization routine. Specifically, ALS decomposes a matrix M as C*S such that M = C*S+E, where E is the error minimized in a least squares approach, M is the measured spectra, C is a scalar component corresponding to the weight of a given spectral contribution, and S is a component spectrum. Spectral decomposition analysis reveals that three spectral components, corresponding to TPEF emission from keratin, NAD(P)H and FAD, are sufficient to describe well (unmodeled variance <0.10) the variations observed in the spectra. From the spectral decomposition, it is clear the NAD(P)H and FAD contributions could be well separated using different excitation wavelengths in combination with spectral emission filtering; however, keratin shows strong spectral overlap with FAD and particularly NAD(P)H at shorter excitation wavelengths. Therefore, we find it necessary to segment the fields into regions of high(+), and low(−) keratin fluorescence, to allow for accurate extraction of the contributions from NAD(P)H, FAD, and keratin, especially when the analysis is based on multi-wavelength but not full spectral images as discussed later. This is achieved simply by applying an intensity-based threshold, incorporating both the 450 and 525 nm detection channels. The relative contributions of NAD(P)H (blue), FAD (green), and keratin (red) to the overall fluorescence emission of the keratin(−) and keratin(+) regions are extracted by spectral decomposition (E–F). Notably, the spectral decomposition confirms the ability of the thresholding technique to remove the keratin contribution, from the superficial and basal layers, with a minimal effect on the relative contribution of NAD(P)H to FAD. This can also be visually observed in the keratin(−) images (iv–vi), where there is a strong reduction of intense yellow pixels corresponding to high intensity in both the 450 and 525 nm detection channels. Since the same threshold is used to analyze all images, in the superficial, highly keratinized layers of the normal tissue equivalents, some keratin fluorescence is present even in the keratin (−) –designated areas. However, since the fluorescence efficiency of keratin is very high, its relative contribution to the overall fluorescence is still high compared to that of NADH and FAD. Nevertheless, this simple procedure works quite well in eliminating keratin fluorescence throughout the depth of the HPV epithelia and most of the layers of the normal ones, so as to allow a simple and fairly robust of NADH and FAD assessment based on the two excitation, two emission wavelength range acquisition. It should be noted that the uppermost, cornified layer in the normal tissue is comprised almost entirely of keratin. The small contributions of NADH and FAD are therefore influenced more by the bleedthrough, ie keratin contribution in the keratin(−) region (Fig. 3E). At 800 nm, keratin is less efficiently excited than at 755 nm and, therefore, the emission spectra predominantly represent NAD(P)H and FAD contributions, even though some keratin fluorescence is still present.

Consistent with the histological and fluorescence images, there is a large spectral contribution from keratin in the cornified layer of the normal tissue as seen by the broadened emission at 755 nm excitation (Fig. 3A), and the increase in the keratin contribution from the spectral decomposition (Fig. 3E–F). At 800 nm, keratin is less efficiently excited than at 755 nm and, therefore, the emission spectra predominately represent NAD(P)H and FAD contributions. In the normal tissue, the fluorescence emission spectral profile at 800 nm excitation shifts throughout the tissue depths indicating changes in the relative contributions of NAD(P)H and FAD (Fig. 3B), and, thus, an enhanced metabolic activity at the basal and suprabasal layers, which is confirmed by relatively lower FAD to NAD(P)H fluorescence ratios extracted from spectral decomposition (Fig. 3E). In contrast, the consistent fluorescence emission profiles from the HPV immortalized tissues indicate that the metabolic activity is relatively constant throughout the depth of the epithelium (Fig. 4B), which is also consistent with the full spectral decomposition analysis (Fig. 4E). As expected, both the normal and HPV-immortalized tissue emission profiles at 860 nm excitation remain constant as a function of depth, since FAD is the dominant fluorophore excited at this wavelength [20], [23].

### Image Analysis for Metabolic Assessment

**Figure 5:**
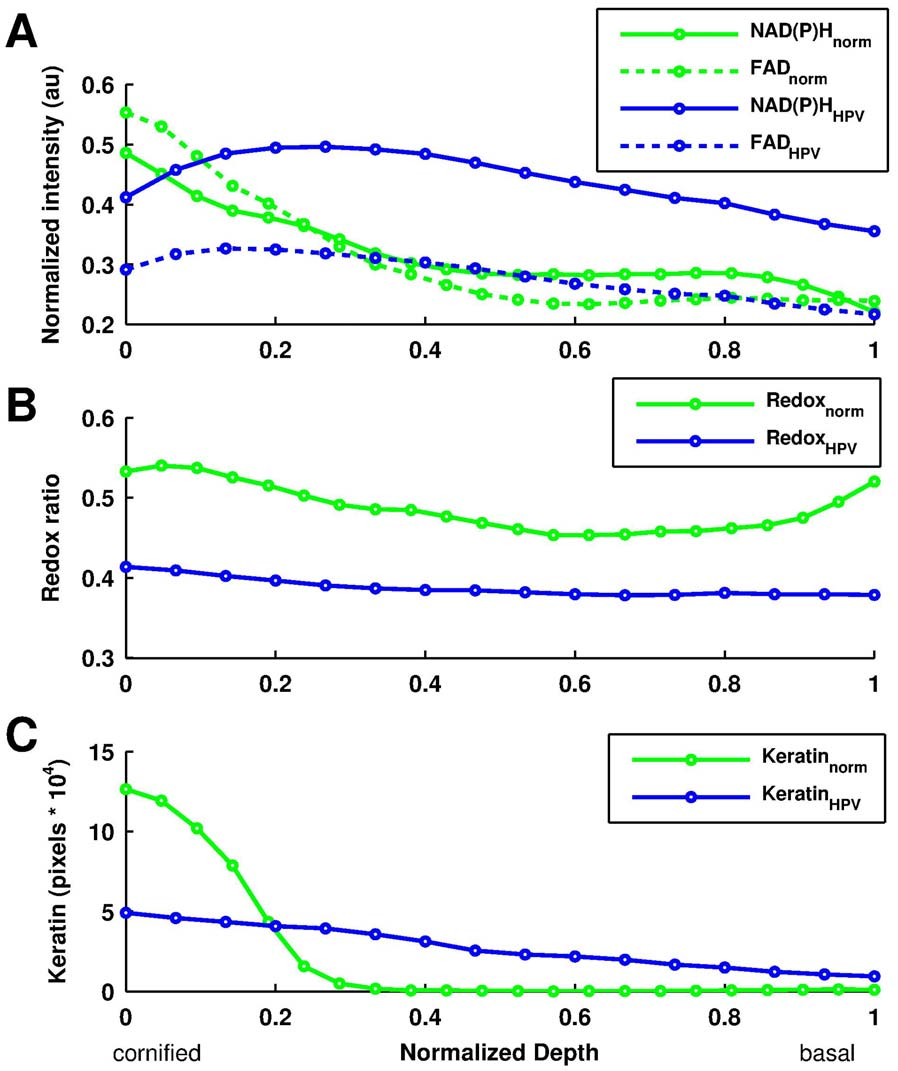
Redox ratio and keratin localization from a representative sample. NAD(P)H (blue) and FAD (red) contribution after being converted to a relative concentration value as a function of depth for normal and HPV-immortalized tissues (a) and the corresponding concentration based redox ratio (b). The keratin contribution is reported by the number of pixels that were removed at each depth increment for the normal and HPV tissue (c).

While spectral analysis is instrumental in identifying the main biochemical contributors to the detected TPEF signals, acquisition of complete spectral images is time consuming and, ultimately, impractical for in vivo studies. Thus, based on the spectral analysis results, we developed a simpler approach to extract the keratin, NAD(P)H and FAD contributions based on analysis of images acquired using 755 and 860 nm excitation at two spectral bands, namely at 455±35 nm and at 525±25 nm. Three independent samples from each tissue type were used for this analysis. The NAD(P)H and FAD intensities from thresholded, keratin(−) areas are shown for single, representative normal and HPV immortalized tissues (Fig. 5A). Representative data are shown from a single specimen, since variations in tissue thickness between and within samples prevent the meaningful assessment of the mean value of this parameter for a specific given depth for all samples; however, depth independent parameters are extracted for multivariate linear regression, as discussed later. Consistent with the spectral decomposition analysis, we find that the metabolic activity, which is inversely correlated with to the redox ratio R = [FAD]/([NAD(P)H]+[FAD]), of the normal tissue increases as we move from the superficial cornified to the deeper tissue layers, and slightly decreases at the lowest layers with a mean redox ratio value of R = 0.481±0.0618 (b). We also find the mean redox ratio variation as a function of depth to be ΔR = 0.00536±0.000350 µm^−1^. In contrast to the normal tissue, the HPV immortalized tissues exhibit lower NAD(P)H and FAD intensities, corresponding to a significant increase (p<0.05) in metabolic rate as indicated by the mean redox ratio (R = 0.379±0.0119). The HPV immortalized tissues also exhibit a significantly more consistent (p<0.05) metabolic depth profile as indicated by the mean redox variability (ΔR = 0.00294±0.00146 µm^−1^).

In conjunction with the redox analysis, we quantify the keratin contribution as a function of tissue depth by assessing the number of pixels in the corresponding keratin(+) areas of the thresholded images (Fig. 5C). While the keratin signal is confined to the uppermost layers of the normal tissue, it is dispersed throughout the HPV-immortalized tissue. Specifically, in the normal tissues 90% of the keratin is confined to the uppermost 26.4±9.0% layers, while the localization of 90% of the keratin in the HPV tissues is confined to the uppermost 80±7.7% layers (p<0.05).

### Multivariate Linear Regression for Improved Diagnostic Power

**Figure 6:**
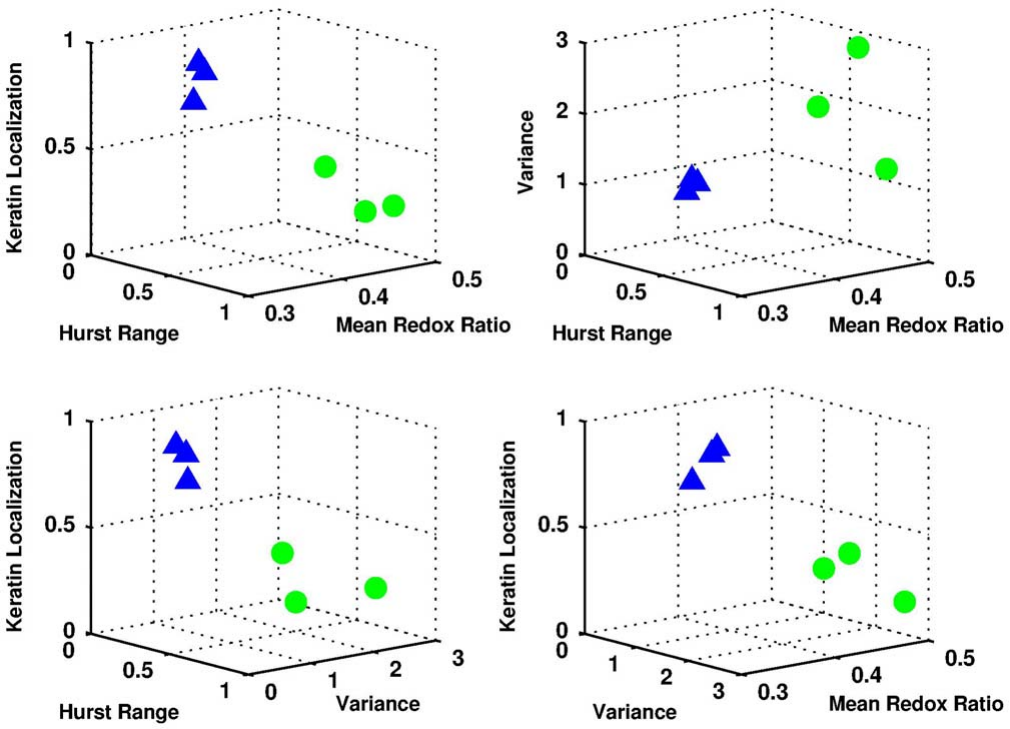
Multi-parametric analysis. Possible combinations of three parameters to discriminate between normal (green circles) and HPV-immortalized (blue triangles) tissues.

To assess the diagnostic power of our multi-parametric approach, we can simultaneously evaluate the organizational, morphological, metabolic, and biochemical parameters as predictors of tissue type. For example, we consider all possible three-parameter combinations for the values of the Hurst parameter range, spatial frequency variance, mean redox ratio, and keratin localization (Fig. 6). Six unique engineered tissues samples (three from each group) were evaluated and characterized using multivariate linear regression. We find that the most diagnostically useful parameter to predict the tissue type is the redox ratio, followed by the Hurst range, spatial frequency variance, and keratin localization, as determined by the type III error. The best model incorporates all four parameters with an R^2^ value of 0.992; however, similar prediction accuracy is obtained using only the redox ratio, Hurst range, and spatial frequency variation due to the notable covariance between the keratin localization and the other parameters.

## Discussion

In conventional screening methods, pathologists typically identify early cancer by assessing morphological markers, such as variation in nuclear size and shape, staining intensity, chromatin pattern, and the appearance of nucleoli or vacuoles and mitotic figures. These methods are highly time-consuming and subjective; furthermore, accuracy is heavily dependent on the expertise of the pathologist [24]. In this study, we present a novel approach for automated detection of early cancer in tissue based on simultaneous quantification of mitochondrial organization, cellular morphology, metabolic activity, and keratin localization from non-invasively acquired TPEF images. This method is capable of assessing morphological changes that are typically evaluated by pathologists, but also allows for quantification of mitochondrial organization and metabolic activity of tissue, which are both potentially valuable diagnostic indicators of disease not available to pathologists using traditional screening methods.

To develop and assess the validity of the analysis methods described in this study, we exploited tissue engineering approaches that allowed us to develop organotypic epithelial tissue cultures that mimic in many respects the biochemistry, architecture and organization of normal and pre-cancerous squamous epithelia [25]. We used the HKc/DR cell line as a model for HPV-associated high-grade premalignant lesions. This clonal line of HPV16 immortalized human foreskin keratinocytes was selected for resistance to differentiation and failure to growth arrest in response to TGF-ß [14], [15]. HKc/DR cells are non-tumorigenic but show patterns of gene expression changes that are similar to cervical carcinoma cells [16]. Consistent with earlier studies that showed that HPV immortalized keratinocytes exhibit histological abnormalities similar to high-grade premalignant lesions [26], organotypic cultures of HKc/DR cells grew in a disorganized fashion and exhibited a low level of differentiation throughout the various depths similar to what was observed in the histological images (Fig. 1B). In contrast, organotypic cultures of primary HFKs exhibited layers of distinct morphology and a keratinized uppermost layer, similar to what is typically seen in healthy tissues. Since the sample is optically sectioned, the tissue remains intact after optical biopsy, and therefore many areas can be interrogated in vivo without inducing pain. The 3-D optical biopsies can be easily viewed from any angle/position in space and quantitatively analyzed using PSD approaches to assess the morphology and mitochondrial organization and multi-wavelength image analysis to assess metabolic activity and keratin localization.

Morphological assessment using PSD analysis is faster and includes more cells than conventional segmentation techniques that have been developed for analysis of stained histological sections [27], [28], and, more recently, for images acquired using non-invasive techniques, such as confocal microscopy [29]–[31]. The goal of many of these studies was to develop methods to rapidly process images and obtain quantitative cellular measures, such as cell border irregularity and nuclear to cytoplasmic ratio, as a means to discriminate between normal and diseased tissue. Although several of these algorithms proved sufficient to identify cells grown in culture, there remains a significant challenge to accurately identify the often ill-defined cell borders in tissue. The result of poor border definition is often the exclusion of many cells from the analysis. To overcome the challenges associated with accurate segmentation, we adopted a Fourier-based approach to assess morphology over many length scales. These methods are sensitive to the spatial TPEF intensity variation patterns from the sub-cellular to the multi-cellular level. Since we are not thresholding or segmenting the images, all cells are included in the analysis.

Our analysis reveals that the lack of variation in nuclear size as a function of depth, which is one of the histopathological hallmarks of intraepithelial neoplasia, is a parameter that can be extracted by examining the variance of the PSD at spatial frequencies that correspond to nuclear sizes. Based on size estimations of the dark appearing areas within the cytoplasm, which should correspond to the non-mitochondria containing nuclei, we have found that the nuclei in the tissues we examine are approximately 8–10 µm in diameter (Fig. 1 E&F insets) and, therefore correspond to the spatial frequencies around 0.1 µm^−1^. As shown in Figure 2, this is a spatial frequency regime of high variance for the normal tissue equivalents, but not for the HPV tissues. In addition, the PSD can be used to assess more subtle and largely unexploited tissue features, such as the level of mitochondrial organization [4]. Assuming self-affine fractal organization, we can extract the Hurst parameter as a quantitative measure of mitochondrial organization to differentiate between normal and precancerous tissue (p<0.05).

There have been a number of studies that use fractal-based characterization for pathological assessment of tissue from the cervix [32], stomach [33], mammary glands [34], breasts [5], [35], [36], and oral cavity [37], [38], while other diagnostic applications of fractal analysis have been reviewed elsewhere [39], [40]. In our work, we calculate the 2-dimensional PSD from label-free images that are not binarized. This is particularly adventageous when alnalyzing tissue images, where the source of contrast is inherently dim endogenous signals, and difficulties arrise when trying to spatially segment (binarize) images based on intensity. In many other studies that use fractal based analyses, excised tissue is stained to enhance the contrast before imaging [32], [34], [35], [37], [38], [41]. With the high contrast agent the images are easily binarized to enhance the cell borders, thus creating a 1-D, self-similar fractal pattern from which the fractal dimension is computed, typically using box counting methods. The 1-D fractal dimension gives insight into the irregularity of cell borders and it has been used to exploit differences between nuclear borders of normal and cervical intraepithelial neoplastic cells [32]. There have also been studies that assess the fractal dimension in conjunction with conventional morphological measurements and report an increased sensitivity over morphological assessment alone [35], [41].

The values of the Hurst parameter used in our analysis can vary between 0 and 1, corresponding to the highest levels of anti-correlation and correlation, respectively, with a Hurst value of 0.5 corresponding to Brownian fractal behavior. The Hurst parameter values that represent the mitochondrial organization of the epithelial cells we examine are consistently lower than 0.5, with the lowest values corresponding to more highly differentiated cells (Fig. 2B). Using similar 2-D, self-affine fractal analysis, Schmitt and Kumar also report anti-correlated fractal patterns from phase contrast images of liver tissue [42]. Also consistent with our findings, Einstein et. al. observe increased levels of anti-correlation of the fractal patterns of chromatin density in breast cell nuclei associated with cancer [5].

In addition to the rich morphological and organization information that can be extracted from analysis of the intrinsic NAD(P)H fluorescence intensity fluctuations, TPEF images acquired at a combination of excitation and emission wavelength bands can be analyzed to acquire quantitative information about metabolic activity and keratin content and localization. To establish the number of components and corresponding spectral emission profiles of the dominant contributors to the detected TPEF images from our tissues, we initially analyzed a series of spectrally resolved images acquired from 400 to 700 nm at 755, 800 and 860 nm excitation. This analysis revealed significant contributions from three chromophores, whose emission spectra were consistent with NAD(P)H, FAD and keratin fluorescence [43]–[45]. Unfortunately, depending on the excitation and emission wavelengths where TPEF is detected, there can be significant spectral overlap between the chromophores, which complicates the extraction of quantitative biochemical information. Guided by analysis of the full spectral emission profiles, we determined that it was important to segment the TPEF images in keratin-positive and keratin-negative regions. Since the fluorescence intensity from keratin is on the order of 3 to 6 times greater than NAD(P)H and FAD [46], we achieved this by a simple thresholding procedure based on the sum of the blue and green detection channel intensities at 755 nm and 860 nm excitation, respectively. We then focused on the keratin(−) pixels and found that in the suprabasal and basal layers we could isolate the contributions of NAD(P)H and FAD from analysis of the images detected by the 455 nm channel at 755 nm excitation and the 525 nm channel at 860 nm excitation with 85–90% confidence, as assessed by comparison with the full spectral decomposition results.

Reliable extraction of the NAD(P)H and FAD fluorescence was important for characterizing the metabolic profile of the normal and dysplastic tissues. In particular, we used the redox ratio, whose value has been shown to be inversely proportional to metabolic activity [7]. An advantage of the redox ratio assessment of tissue compared to individual estimates of NADH or FAD is that artifacts potentially introduced by absorption of chromophores, such as keratin, are minimized, since they similarly affect NADH and FAD. We find that the redox ratio decreases as we move from the superficial/cornified to the suprabasal cell layers of the normal epithelial tissues and then it increases slightly as we reach the basal layer. In comparison, we observed significantly less change in the redox ratio depth profile of the HPV-immortalized tissues (p<0.05), consistent with the apparent loss of differentiation. Such differences in the depth-dependent variation of metabolic activity between normal and pre-cancerous tissues have been reported by other investigators [10], [45], [47]–[51]. Furthermore, we report that the overall metabolic activity is higher in the pre-cancerous tissues than in normal tissues (p<0.05), consistent with previous studies [46], [48], [52]–[55]. More recently, researchers observed that the redox ratio variations are also correlated with the degree of metastatic potential and location within the tumor [56].

While elimination of the fluorescence contribution from keratin was necessary to accurately assess metabolic activity, we also used the keratin localization as a means to discriminate between tissue types (p<0.05). In the normal tissues, we found that keratin, likely type 10 and 13 from terminally differentiated cells [45], [49], was restricted to the upper layers and consistently expressed throughout the depths of the HPV tissues. Indeed, keratin expression has been correlated with cervical cancer progression [57]–[62], with keratin 17 prominently expressed at various tissue depths of dysplastic tissue [57], [61]. Due to the high quantum yield of keratin [20], it could serve as an additional diagnostic biomarker than can be much more easily extracted from analysis of intrinsic TPEF compared to conventional histological images, where identifying keratin amongst cells is not trivial [57].

The biochemical, morphological and organizational information extracted from analysis of the intrinsic TPEF images is complementary and can be used in combination to ultimately develop highly sensitive and specific algorithms for the detection of pre-cancerous lesions. Using multivariate linear regression, we found that such a combination provided a better means to identify precancerous changes in tissue when analyzed simultaneously. We specifically evaluated and considered the Hurst range (p<0.05), redox ratio (p<0.05), keratin localization (p<0.05), and variance of the PSD (p<0.10). Addition of the keratin localization did not improve significantly the diagnostic potential over the combined use of the other three parameters in the small sample size of this study. It is important to note, however, that these studies are to be regarded as a proof of principle, where we harnessed organotypic tissue culture of a clonal HPV16 immortalized cell line that was previously established as a model system for HPV-associated high-grade premalignant anogenital tract lesions. To establish the true diagnostic potential of the parameters that we derived from this study, it will be important to validate these on human tissue samples, where a much more heterogeneous cell population will be expected. Most premalignant lesions contain areas with different levels of cellular abnormalities. Low-grade lesions, in particular, often retain at least some ability to undergo differentiation. Improvements in diagnostic accuracy using a combination of morphological and biochemical information extracted typically through the use of multiple optical modalities has been observed previously, consistent with our findings [9], [10], [52], [63], [64]. A unique aspect of the approach presented in this study is that all the information is extracted from analysis of a single type of imaging with approaches that can be implemented in real time. In addition, the procedures include all cells in a given field and require no human input, unlike commonly used segmentation approaches. Since the methods are based on non-invasive measurements, the algorithms could be useful in a screening/diagnostic device that can assess appreciable tissue areas and detect the early microscopic changes associated with pre-cancer development relying on information that clinicians do not currently have access to.

## Supporting Information

Figure S1 **Normalized component spectra used in ALS algorithm.** FAD (green) and NAD(P)H (blue) emission spectra which were found to be similar irrespective of excitation wavelength. Keratin (red) emission spectra from 755 nm (dashed), 800 nm (dotted), 860 nm (dot/dash) excitation wavelengths.
(TIF)
