# Supplementary figures and images for: Automated Biochemical, Morphological, and Organizational Assessment of Precancerous Changes from Endogenous Two-Photon Fluorescence Images
Source: PLoS One. 2011 Sep 9;6(9):e24765. doi: 10.1371/journal.pone.0024765 (PMC3170385; doi:10.1371/journal.pone.0024765)

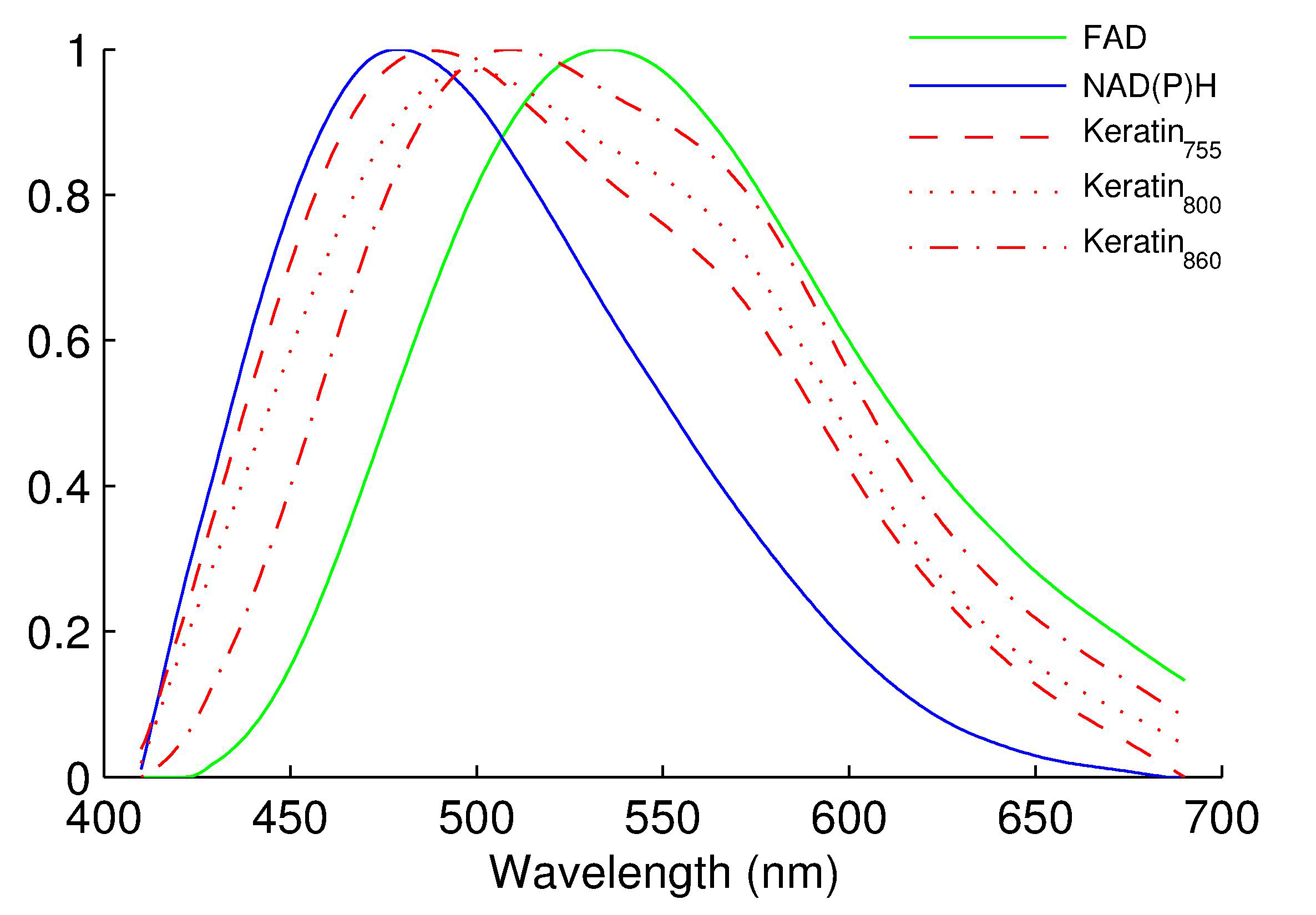

Supplement: Figure S1 — Normalized component spectra used in ALS algorithm. FAD (green) and NAD(P)H (blue) emission spectra which were found to be similar irrespective of excitation wavelength. Keratin (red) emission spectra from 755 nm (dashed), 800 nm (dotted), 860 nm (dot/dash) excitation wavelengths. (TIF) [file pone.0024765.s001.tif]
